# A novel high performing multiplex immunoassay Multi-HTLV for serological confirmation and typing of HTLV infections

**DOI:** 10.1371/journal.pntd.0009925

**Published:** 2021-11-01

**Authors:** Lola Marqué, Peter Liehl, Jasper De Boer, Hans Pottel, Edward L. Murphy, Roberta Bruhn, Mars Stone, Zhanna Kaidarova, Tzong-Hae Lee, Michael Busch, Maan Zrein

**Affiliations:** 1 InfYnity Biomarkers, Lyon, France; 2 Department of Public Health and Primary Care, KU Leuven Campus Kulak Kortrijk, Kortrijk, Belgium; 3 Vitalant Research Institute, San Francisco, California, United States of America; Escola Bahiana de Medicina e Saúde Pública, BRAZIL

## Abstract

**Background:**

Human T-Cell Lymphotropic Viruses (HTLV) type 1 and type 2 account for an estimated 5 to 10 million infections worldwide and are transmitted through breast feeding, sexual contacts and contaminated cellular blood components. HTLV-associated syndromes are considered as neglected diseases for which there are no vaccines or therapies available, making it particularly important to ensure the best possible diagnosis to enable proper counselling of infected persons and avoid secondary transmission. Although high quality antibody screening assays are available, currently available confirmatory tests are costly and have variable performance, with high rates of indeterminate and non-typable results reported in many regions of the world. The objective of this project was to develop and validate a new high-performance multiplex immunoassay for confirmation and discrimination of HTLV-1 and HTLV-2 strains.

**Methodology/Principal findings:**

The multiplex platform was used first as a tool to identify suitable antigens and in a second step for assay development. With data generated on over 400 HTLV-positive blood donors sourced from USA and French blood banks, we developed and validated a high-precision interpretation algorithm. The Multi-HTLV assay demonstrated very high performance for confirmation and strain discrimination with 100% sensitivity, 98.1% specificity and 100% of typing accuracy in validation samples. The assay can be interpreted either visually or automatically with a colorimetric image reader and custom algorithm, providing highly reliable results.

**Conclusions/Significance:**

The newly developed Multi-HTLV is very competitive with currently used confirmatory assays and reduces considerably the number of indeterminate results. The multiparametric nature of the assay opens new avenues to study specific serological signatures of each patient, follow the evolution of infection, and explore utility for HTLV disease prognosis. Improving HTLV diagnostic testing will be critical to reduce transmission and to improve monitoring of seropositive patients.

## Author summary

HTLV viruses are responsible for more than 10 million cases of infection worldwide. The infection is considered as a neglected disease due to lack of vaccines and treatments. Accurate diagnosis is crucial for counselling infected persons and prevention of secondary transmissions. In spite of the development of excellent serological screening assays, many cases of indeterminate and untyped results are still regularly reported and their infection status remain uncertain. To address the need of more precise diagnosis, we have developed a new cutting-edge in-vitro diagnostic confirmation test, named Multi-HTLV, which has been validated on a large panel of HTLV samples. The test is a multiplex immunoassay allowing powerful detection of antibodies against HTLV through combination of a set of selective and validated virus-specific antigens in a blood sample. The Multi-HTLV assay increases the reliability of HTLV diagnostics and strain typing thanks to a high precision mathematical algorithm.

## Introduction

Human T-cell lymphotropic viruses (HTLV) were the first human retroviruses to be discovered. They cause blood infections through three transmission routes: mother-to-child transmission (mainly associated with prolonged breast-feeding), sexual transmission and contaminated blood products (blood recipients and drug users) [1]. HTLVs are divided in two major strains, HTLV-1 and HTLV-2, and two novel strains recently discovered in Cameroon, HTLV-3 and HTLV-4 [2–4]. HTLV infections are mainly caused by HTLV-1 with an estimated 5 to 10 million infections worldwide [1]. HTLV-2 infections are roughly 6 to 12 times less prevalent than HTLV-1 [5]. Both strains share 60% sequence homology, and differ in their epidemiology, pathogenicity and clinical manifestations [6].

HTLV-1 is divided into four subtypes and circulates mainly in Japan, the Caribbean, West and Central Africa, South America and Australo-Melanesia [1,7]. HTLV-1-infected individuals have a 3–7% risk of developing two severe diseases, namely adult T-cell Leukemia/Lymphoma (ATLL) and HTLV-1 associated myelopathy/Tropical Spastic Paraparesis (HAM/TSP) [8]. HTLV-1 infection is also associated with increased overall morbidity and with the development of other pathologies such as uveitis, dermatitis, respiratory disorders, infectious diseases and cancer [9,10]. HTLV-2 is endemic among indigenous American people, pygmies in Africa and intravenous drug users in urban areas of the US, Europe and Latin America [11,12]. HTLV-2 is not associated with well-defined clinical manifestations, but some studies have shown that it can occasionally be associated with cutaneous lymphoma, chronic myelopathy, neurological syndromes such as HAM/TSP and may increase overall cancer morbidity [13–15]. All HTLV-associated syndromes are currently considered as neglected diseases, because neither a vaccine nor curative treatment is available [16]. Prevention of HTLV infection is currently based only on the restriction of transmission and can be achieved by assessing the infection status of persons in endemic regions, tracking all HTLV-infected individuals, and through clinical management of HTLV-associated syndromes [17]. Worldwide, many people are underdiagnosed, mainly due to asymptomatic form of infection, long latency of clinical manifestations after infection, and the lack of knowledge of many healthcare workers and systematic screening [18]. For the diagnosis of HTLV infection, each region of the world applies its own screening algorithm. Typically, a serological screening assay is first used to detect antibodies which reflect chronic infection, using an Enzyme-Linked Immuno-Sorbent (ELISA), Chemiluminescent or Particle agglutination assays [19]. If the result is reactive, confirmation is performed using an immunoblot assay, predominantly Western Blot (WB) or line immunoassay (INNO-LIA), according to currently used algorithms of each country; Polymerase Chain Reaction tests (PCR) can also be used to detect and genotype viral DNA in lymphocytes, although no PCR tests are commercially available. The WB and INNO-LIA are the most commonly used tests for confirmation. The assays are based on antibodies binding to HTLV-I and HTLV-II *Gag* and *Env* proteins derived from viral lysates or recombinant proteins. Many studies have revealed shortcomings of the WB technique. Indeed, in all regions of the world and specially in high risk populations, a high number of indeterminate and non-typable results have been found [20,21]. The large number of unclear results hinders correct diagnosis and can cause problems for counselling by public health, blood bank and organ transplantation organizations that conduct screening [22]. Some countries such as Brazil intend to replace WB with PCR tests for confirmation, which offers higher sensitivity and more accurate strain discrimination [20,23]. However, the PCR methods can lack sensitivity due to the low proviral load in lymphocytes circulating in the blood of HTLV-infected individuals and in some cases of co-infection (e.g. HTLV-2/HIV co-infection) due to proviral load reduction [24]. Therefore PCR should not be used alone for confirmation but rather in combination with WB or for investigation of inconclusive results [25,26]. The INNO-LIA immunoassay, widely used in Europe, is currently considered to be excellent for serological confirmation [20,27,28]. The test is superior to WB, due to clearer interpretation criteria directly linked to a decreased number of antigens, readability, and improved performance by eliminating uncertain and false positive results [20,26,29]. However, the test is costly, making it difficult to use in routine diagnostics, especially in resource limited areas such as South America [20]. Furthermore, the INNO-LIA assay has not upgraded its antigen composition to take into consideration different genotypes of HTLV-1 or newly discovered HTLV types within the last two decades [4,30]. Access to a new assay that improves confirmation and strain typing in high-risk populations is considered as key strategy to improve counselling and infected patient management and reduce secondary disease transmission [16].

The aim of this study was to develop a new high performing and cost-effective multiplex test to confirm HTLV infection and discriminate the two strains, HTLV-1 and HTLV-2. We used a microplate-based multiplex platform to identify the most relevant antigens (recombinant proteins and synthetic peptides) and developed a readable and easy-to-use assay. The developed assay with updated high-performance antigens offers improved confirmation of HTLV infection and can be performed in routine laboratories with standard lab equipment.

## Methods

The methods section was described in accordance with the Standards for Reporting of Diagnostic Accuracy Studies (STARD) protocol (S1 Appendix).

### Ethics statement

Written informed consent was obtained from all participants in the United States-based cohort HTLV Outcomes Study (HOST), including use of frozen biospecimens for future research. The study was approved by the University of California San Francisco Committee on Human Research (IRB#s H5901-14117-13D & 11–05747) and other institutional review boards. Written informed consent was obtained by EFS from blood donors after a detailed explanation of how their donation would be used for research purposes.

### Study populations and sample origins

For this study, different sample collections were used (Table 1).

**Table 1:** Summary of sample collections characteristics.

Sample collection	Sample type	N
HOST, USA	HTLV-1	97
	HTLV-1/HCV	3
	HTLV-2	87
	HTLV-2/HCV	12
	Negative Blood Donor	198
EFS, France	HTLV-1	111
	HTLV-1/HBV	14
	HTLV-1/HBV/*Plasmodium*	5
	HTLV-1/HBV/HCV	1
	HTLV-1/*Plasmodium*	1
	HTLV-1/HBV/*T*. *Pallidum*	1
	HTLV-2	6
	HTLV-2/HBV/HCV/ *Plasmodium*	1
	Negative Blood Donor	28
Trans-Hit Bio, Canada	HTLV-2	65

HOST, United States-based cohort HTLV Outcomes Study; EFS, Etablissement Français du Sang; N, Number; HBV, Hepatitis B virus; HCV, Hepatitis C virus.

### HOST cohort (USA)

This prospective study was conducted with samples from the United States-based cohort HTLV Outcomes Study (HOST) which includes samples from subjects recruited from five major US blood donation centers [31,32]. Based on eligibility criteria, the cohort originally included a total of 1340 participants, of whom 154 were HTLV-I-infected, 387 HTLV-II-infected, and 799 were uninfected blood donors. All subjects first made one blood donation (between 1990 and 1992) and some of them were followed-up for almost 20 years. Enrollment into the cohort was based upon screening by a licensed HTLV-I viral lysate ELISA assay (Abbott, USA), followed by WB confirmation (Abbott, USA). Then, each participant was recalled for the study evaluation and an in-house PCR for HTLV-1 versus HTLV-2 typing was performed, followed by recombinant p21e EIA (Cambridge Biotech, USA) and recombinant p21e WB (Cambridge Biotech, USA) if PCR was negative [31]. If PCR and other supplemental serological results were discordant, donors were recalled and retested using fresh blood samples. At every visit, cohort participants were interviewed in detail for symptoms, followed by a physical and neurological examination [33]. In the frame of another clinical trial at CTS Laboratories an additional confirmatory antibody test was performed with the INNO-LIA HTLV-I/II (Fujirebio, Japan). Proviral load was monitored by quantitative PCR using type-specific primers [34]. For some patient’s, symptoms, risk behaviors, physical examinations results and co-infections were available [33].

**Fig 1:**
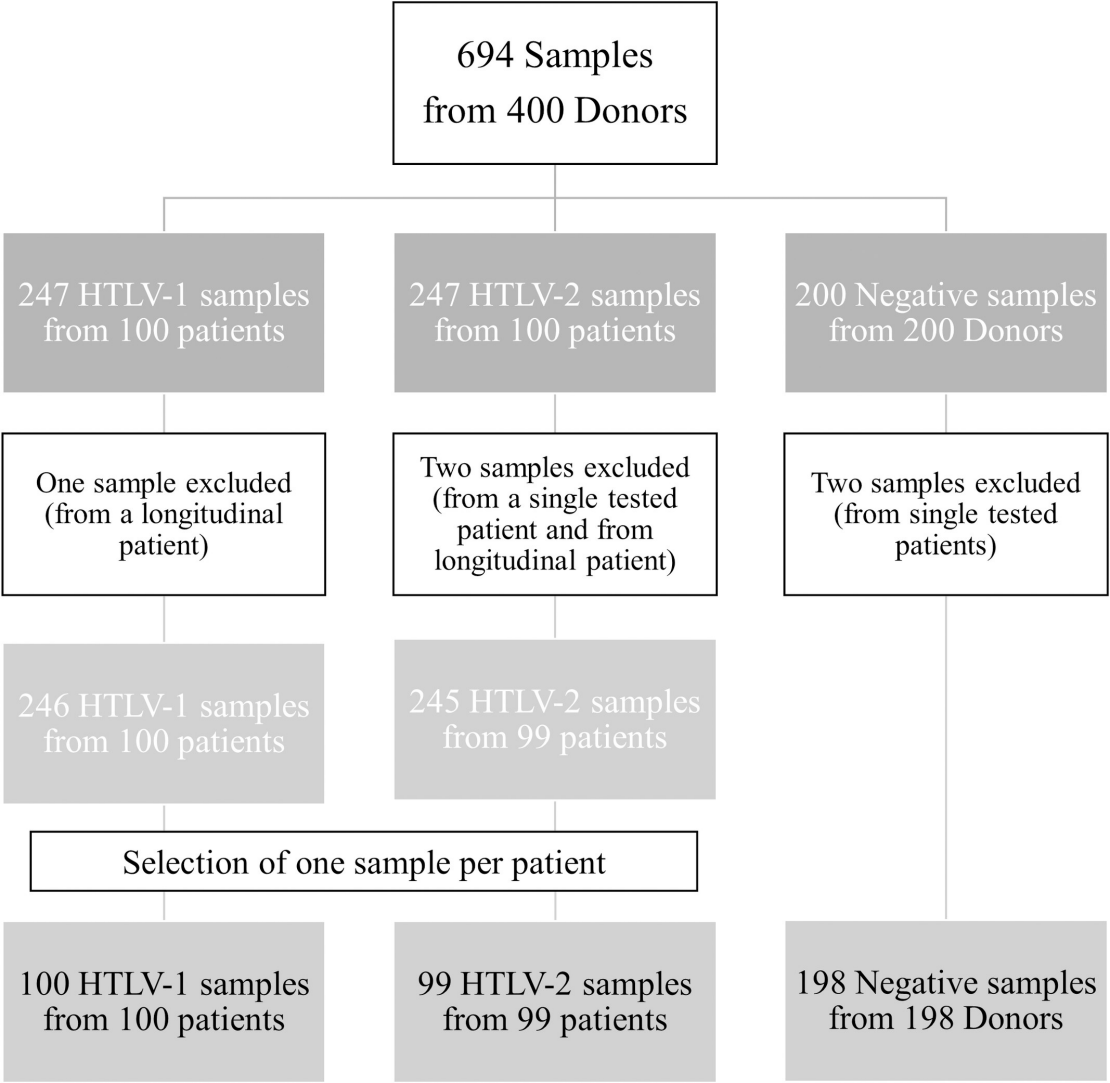
HOST Cohort description. The tree represents the distribution of the number of samples within the HOST cohort within the three categories of negative, HTLV-1, and HTLV-2 patients.

For the current study, 694 frozen serum specimens were obtained from the cohort repository. We excluded from the study three samples because of labeling errors and two samples because of mismatches, which could lead to misinterpretation. A total of 689 samples from 397 participants were therefore eligible for the analysis. Among them, 60 HTLV-positive patients were followed over time in a longitudinal study and tested to analyze the evolution of their serological profile. However, in order to have independent data for each HOST participant, we included in the study only the first sample tested of each participant, more specifically, 199 HTLV-positive samples from 199 participants (100 HTLV-1 from 100 patients and 99 HTLV-2 from 99 patients) and 198 negative samples from 198 participants (Fig 1; S2 Appendix).

### Etablissement Français du sang EFS (France, Tours)

We obtained 140 positive samples (133 HTLV-1 and 7 HTLV-2) and 28 seronegative samples from different French blood donations (transfer agreement 20120224). Samples were collected between 2008 and 2013, and screening was performed by ELISA, using either Prism HTLV-I/HTLV-II or Architect rHTLV-I/II assay (Abbott, USA), and then HTLV positive samples were confirmed and typed by INNO-LIA HTLV-I/II.

### Trans-Hit Bio (Canada)

We obtained 65 HTLV-2 samples from a commercial source. All patients came from USA and had available data on ethnicity and ages. Samples were collected between 2013 and 2016, screened with an HTLV-1/2 ELISA (brand not specified by Trans-Hit Bio) and subsequently confirmed as HTLV-2 positive by WB (brand not specified by Trans-Hit Bio).

### Multi-HTLV assay

The multiplex technology allows multiple parameters to be combined in a single well of a 96-well microplate. The printing process is based on non-contact piezo electric impulsion of a defined volume of an antigenic solution. We used the SciFLEXARRAYER printing system (SCIENION, Germany) to print HTLV antigens, at the bottom of each well at precise X-Y coordinates, under controlled humidity and temperature parameters. This technology was used first to make a selection of the most relevant antigens and second for the design and production of the assay, which has already been used in this manner for the development of other confirmatory test for Chagas disease [35]. Each antigen spot is printed in duplicate and corresponds to a specific antigen composition.

The Multi-HTLV assay is composed of (i) three confirmatory antigen spots for binding specific HTLV-1 and HTLV-2 antibodies and derived from common-type and type-specific immunodominant epitopes of *env* GP21, *env* GP46 and *gag* P19; and (ii) three discriminatory antigen spots derived from type-specific immunodominant epitopes of HTLV-1 *env* GP46, HTLV-1 *gag* P19 and HTLV-2 *env* GP46. In contrast to other confirmatory immunoassays, we excluded the detection of anti-P24 antibodies in the Multi-HTLV test because it has been shown in WB assays that indeterminate samples frequently show an isolated P24 band attributable to false reactivity [36]. Relevant antigens were designed and obtained synthetically according to the reviewed and published non-redundant sequences available at the repository www.uniprot.org (Table 2).

**Table 2:** Antigen’s description.

Antigen / Type specificity	Accession Number
*env* GP21 / HTLV-1 & HTLV-2	P03383
*gag* P19 / HTLV-1 & HTLV-2	Q9WI14
*gag* P19 / HTLV-1	P14077
*env* GP46 / HTLV-1 & HTLV-2	P23064
*env* GP46 / HTLV-1	P23064
*env* GP46 / HTLV-2	P03383

Accession number of antigens included in the Multi-HTLV assay.

**Fig 2:**
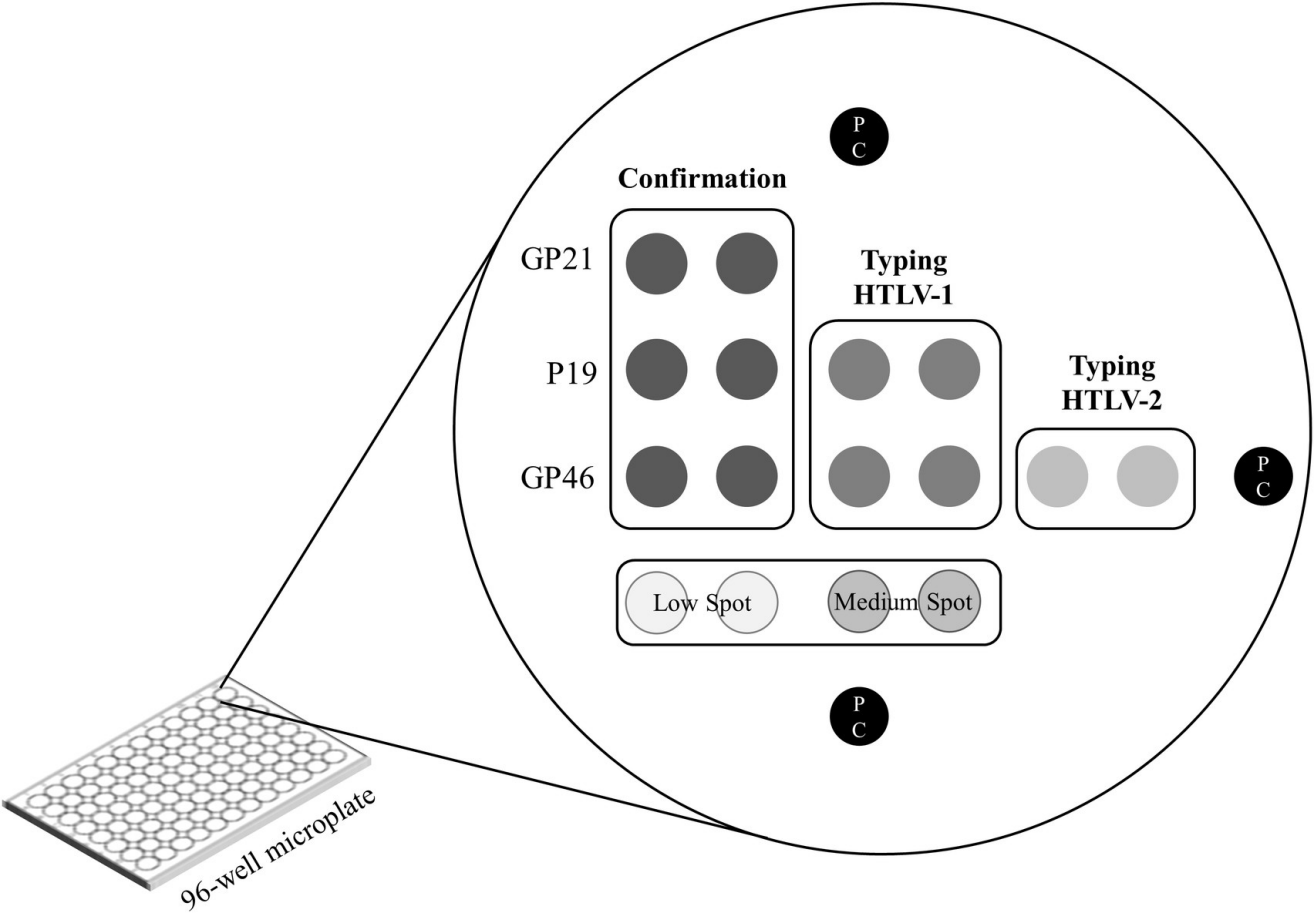
Matrix design of Multi-HTLV assay. This matrix is printed in each well of the 96-well microplate and contains three confirmatory antigen spots, as well as two HTLV-1 strain typing spots and one HTLV-2 strain spot. Each probe was printed in duplicate. The “low”, “medium” and positive controls spots are added for visual interpretation, orientation and as a control of the test steps.

Each antigen composition was individually optimized to obtain the best performance in terms of sensitivity and specificity, and by calculating the areas under the Receiver Operating Characteristic curve (AUC), using SPSS Statistics software (IBM, USA, version 19.0). This optimization step was performed by using a panel of HTLV-infected and seronegative samples from EFS. Further, three positive control (PC) spots were printed in each well to define a precise spatial orientation pattern and to validate the correct sequential addition of all reagents (human serum samples, enzyme conjugate and substrate). To facilitate the visual interpretation at the end of the test, two additional control “low”- and “medium”-spots–composed of anti-human-IgG antibodies–were printed and indicate the "minimal" or "intermediate" reactivity for each patient (Fig 2).

Following the spotting process, the plates were incubated overnight to allow coating of the antigens at optimal conditions of humidity and temperature. Then, they were dried at 37°C for 2 hrs and washed with PBS-T (phosphate-buffered saline with 0.05%-Tween 20). A blocking solution containing Bovine Serum Albumin (BSA) was added into the microplate for 2 hrs at room temperature (RT) to avoid nonspecific binding during the testing process. After the saturation step, the microplates were then washed with PBS-T. For long term storage, the blocked microplates were dried at 37°C during 2 hrs, then stored at 4°C in sealed plastic bags with desiccants.

The Multi-HTLV test is performed very similarly to a standard ELISA assay enabling simultaneous detection of circulating antibodies to a set of selective and validated antigens. Hundred μL of diluted patient samples in PBS-T (0.05%, pH 7.5) and BSA (1%) (1:50) was added to each well, then microplate was incubated for 1 hr at RT. After three washing steps with PSB-T, 100 μL of anti-human-IgG secondary antibody conjugated to Horseradish Peroxidase, diluted in a defined volume of PBS-T (0.05%, pH 7.5) and BSA (1%) buffer (1:2000), was added and the microplate incubated for 1 hr at RT. To remove unbound secondary antibodies the microplate was washed three times with PSB-T. For the detection of conjugated enzyme activity, a precipitating substrate solution (3,3′,5,5′-Tetramethylbenzidine) was added and the microplate incubated in the dark for 20 min at RT. Upon contact of the substrate with the enzyme, it is converted to an insoluble permanent dark-blue precipitate. The reaction was stopped by removing the substrate solution, and drying the microplates at 37°C for 10 min. Each plate was read and analyzed both visually and using a specific microarray reader sciREADER CL2 (SCIENION, Germany) which acquires high-resolution digital images and an integrated software calculates the pixel intensity for each spot. We calculated the mean value of the duplicated spots to get the net intensity of each antigen. The data were incorporated into a Microsoft Excel 2019 database and analyzed using a specific Multi-HTLV interpretation algorithm to determine the final result (see section "Interpretation algorithm").

### Statistical analysis

#### Dataset

Data from three cohorts (HOST, EFS, Trans-Hit Bio) were pooled. Gold standard HTLV type assignments were available for the HOST cohort samples by either PCR or type specific ELISA and INNO-LIA results. For the two other studies, sample type assignment was only available based on INNO-LIA test results. The pooled dataset was split into a development and validation set. All data from EFS and Trans-Hit Bio were allocated to the development set and the HOST samples were allocated randomly (p = 0.5) to either the development or the validation dataset. Only HOST samples were included in the validation set because of PCR-based HTLV classifications. PCR has high confirmation power and strain discrimination, which in turn leads to more accurate validation analysis. The validation set consisted of 96 positive samples (53 HTLV-1 and 43 HTLV-2) and 103 negative samples.

The addition of EFS and Trans-Hit Bio data to HOST data for the development set allowed a larger sample size, which is beneficial for statistical methods and machine learning algorithms. It comprised 308 positive samples (180 HTLV-1 and 128 HTLV-2 samples) and 123 negative samples. For both HTLV detection and typing, a logistic regression (LR) model was trained. Outliers were expected in the development set due to possible imperfect INNO-LIA labeling of EFS and Trans-Hit Bio samples. Sample deviances were used to detect outliers, which were omitted if the human interpretation of the Multi-HTLV assay did not match the INNO-LIA label. Due to the gold standard labeling of HOST samples, only samples with deviances higher than the maximum deviance of HOST samples were checked. Deviances below this threshold were expected to arise from regular variability but not necessarily from mislabeling.

#### Interpretation algorithm

Logistic regression was used as a baseline positive/negative classification method using GP21-I/II (Confirmation), P19-I/II (Confirmation), GP46-I/II (Confirmation), P19-I, GP46-I and GP46-II as independent variables (features). To prevent (quasi-) complete separation problems, Bayesian analysis with non-informative prior assumptions was applied, by use of the R *arm* package (version 1.11–2) [37]. A backward selection procedure was used to remove non-significant features, based on Akaike’s information criterion, to achieve the final minimal adequate model [38]. A Random forest model was also examined but no performance improvements were found. This analysis is therefore not included in this article. We have combined an additional algorithm to logistic regression formulas, to categorize questionable samples into an indeterminate category. First, a minimal intensity value of 10 was determined for labeling confirmation antigens as positive. A 2-cluster Gaussian mixture model was fitted using the R mclust package (version 5.4.7), and the clusters’ intersection point was used as threshold.

**Fig 3:**
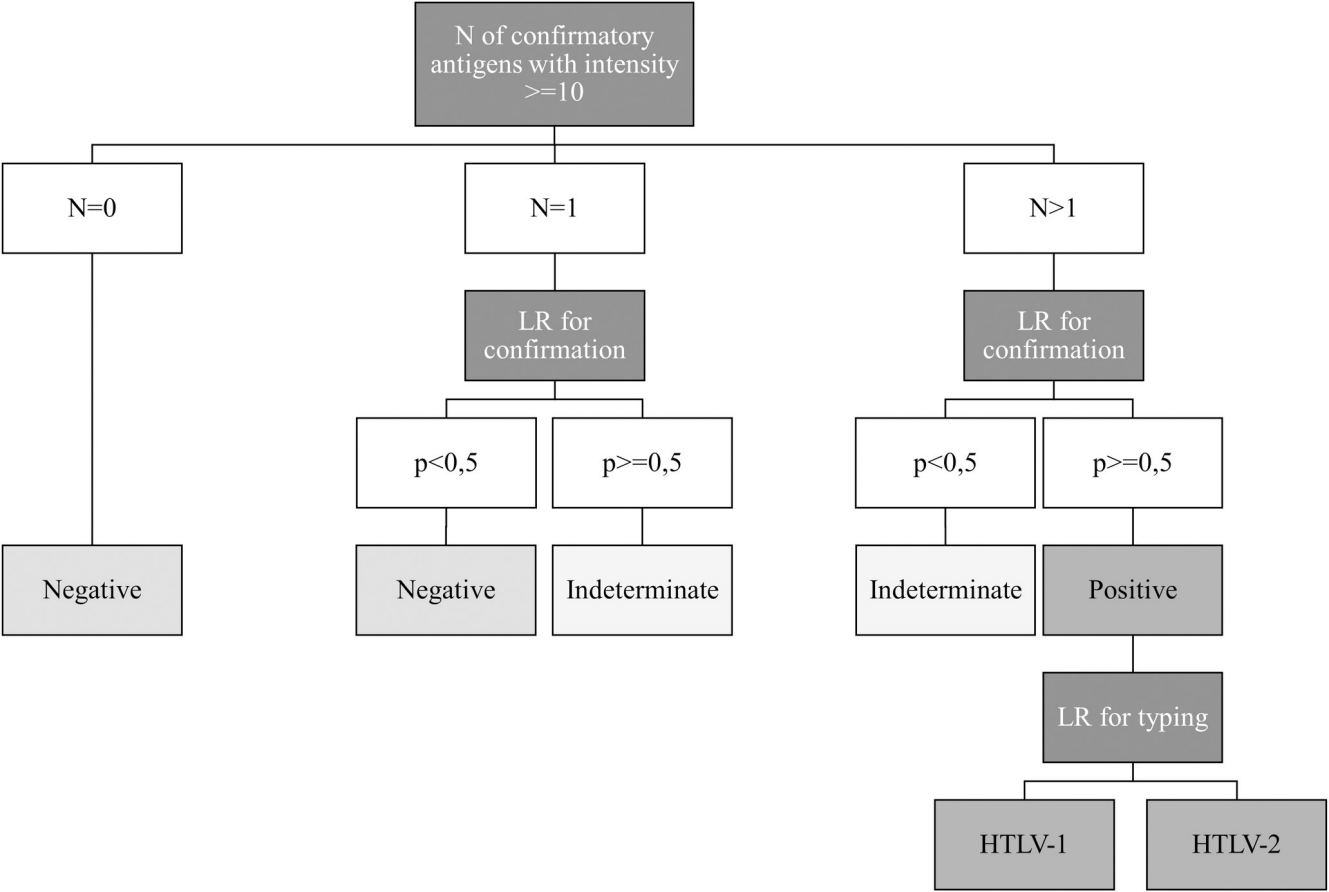
Simplified algorithm model for Multi-HTLV confirmation result. The decision tree representing the interpretation algorithm of the Multi-HTLV test, is divided into three steps. The first step is to classify the sample into three categories according to the number of confirmatory antigens with an intensity greater than or equal to 10. The second step is to apply the first logistic regression formula for confirmation to categorize samples with a number of reactive antigens greater than or equal to 1. Finally, the third step is to apply a logistic regression formula for typing, only on positive samples.

Then, we developed an algorithm based first on counting the number of positive antigens per sample and then applying logistic regression formulas. The algorithm is as follows: if the number of confirmatory antigens is zero, then the sample is directly considered to be negative; if the number of antigens is equal or higher than one, then a first logistic regression formula for confirmation is applied. It permits classification of samples with one reactive antigen among negative (LR probability (p)<0,5) or indeterminate categories (p> = 0,5), and samples with number of antigens higher than one in indeterminate (p<0,5) or positive (p> = 0,5) categories. Thereafter, only positive samples are typed as HTLV-1 and/or HTLV-2 by applying a second logistic regression formula (Fig 3). The Interpretation Algorithm was implemented in Microsoft Excel 2019 for further data processing.

#### Performance analysis

After application of the interpretation algorithm, sensitivity (number of Multi-HTLV-positive values divided by number of PCR-positive samples) and specificity (number of Multi-HTLV-negative values divided by number of PCR-negative samples) with corresponding confidential interval (CI) of 95%, were used for diagnostic accuracy and performance evaluations of the Multi-HTLV assay. Indeterminate results obtained were included for the calculation of sensitivity and specificity. In this case, a PCR-negative sample that was indeterminate with Multi-HTLV, was treated as a false positive. Conversely, a PCR-positive sample that was indeterminate, it was counted as a false negative.

## Results

**Fig 4:**
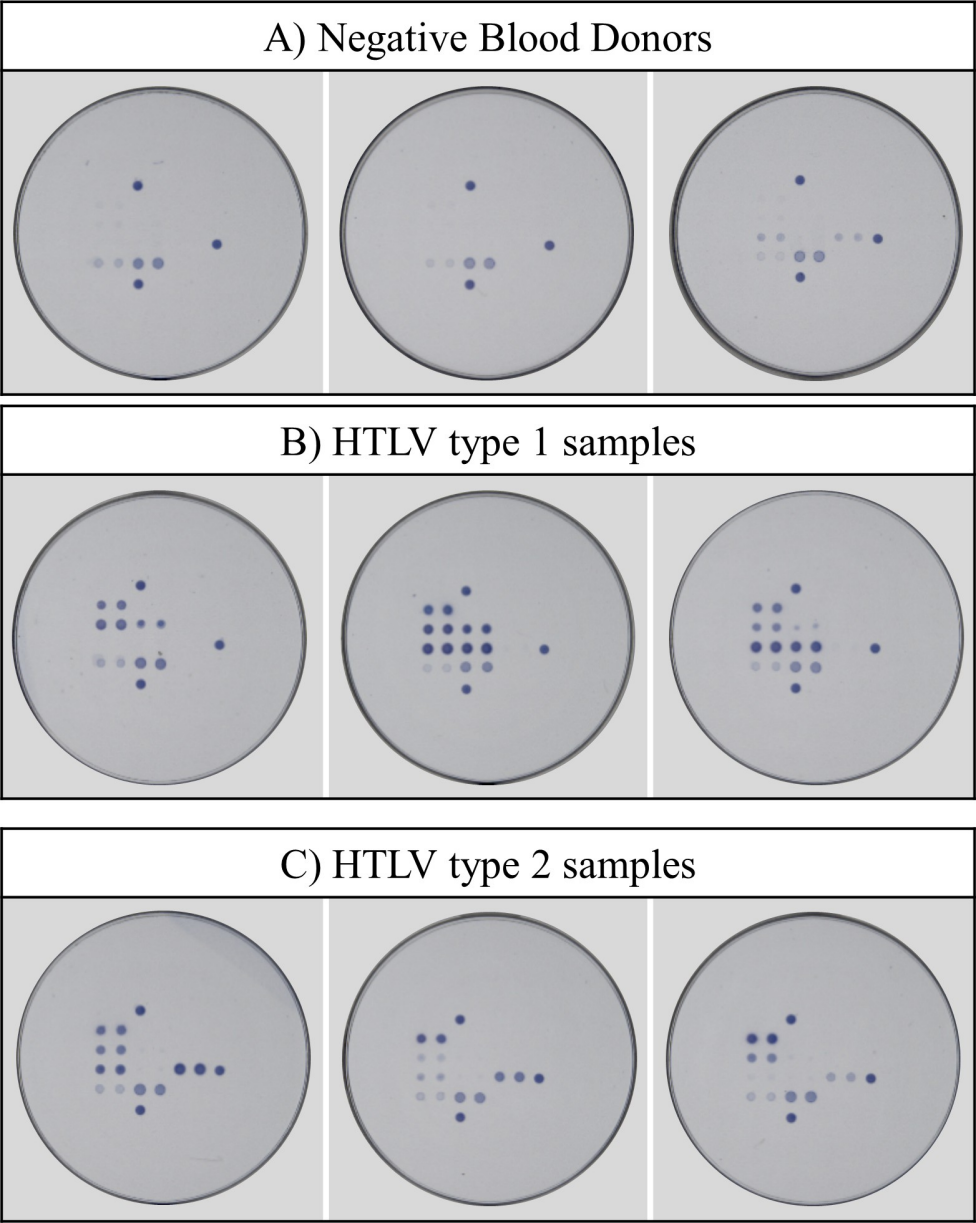
Examples of serological patterns obtained with Multi-HTLV. (A) Examples for three negative blood donors. These are either non-reactive samples or samples that show cross-reactivity with isolated GP46 confirmatory and/or GP46-II typing antigens. (B) Examples for three HTLV-1 patients. Different profiles were observed depending on the individual immune response of each patient. (C) Examples for three HTLV-2 patients. Different profiles were observed depending on the individual immune response of each patient.

First, we evaluated the Multi-HTLV confirmatory assay by analyzing the immunoreactivity of each antigenic marker for all tested patients. By visual analysis of images recorded with a specific image reader, we could clearly separate the majority of HTLV negative and positive results. Next, a mathematical algorithm was developed to facilitate a faster and accurate analysis of the confirmation and typing results. In addition, the algorithm enables precise determination of atypical profiles, for which visual interpretation is more complicated (S3 Appendix). The training of the algorithm was performed with the development set and then applied to the validation set. Each sample was confirmed by classification using the net intensities of the three confirmatory antigens and then by applying the two logistic regression formulas for confirmation and typing. Because HOST samples were tested and typed by both PCR and INNO-LIA HTLV, for sake of clarity, we describe here the results of this fully documented cohort. The performance of the algorithm is presented for both the development and validation datasets to evaluate the reactivity of all samples of the HOST cohort. However, the strength of the algorithm can only be evaluated on the validation dataset, since it was not used to develop the algorithm. Sensitivity and specificity were calculated based on perfect matching of the results as compared to PCR gold standard. We obtained a sensitivity of 100% (96,4–100 development set; 96,15–100 validation set) in both sets and a specificity of 97,90% (92,65–99,42) and 98,06% (93,19–99,47) for the development and validation sets, respectively. The proposed new method achieves slightly improved performances compared to INNO-LIA in terms of reduced indeterminates (4 vs. 5). Noteworthy, all the patients displayed different serological patterns depending on the HTLV-type and individual immune reactivities (Fig 4).

By analyzing the classification of the samples compared to the PCR gold standard, the specificity did not attain optimal performance as two negative samples in each set were classified in the indeterminate category (Table 3).

**Table 3:** Analysis of the Multi-HTLV results on the HOST cohort.

Gold Standard Results	Multi-HTLV Results								Total
	Negative		HTLV-1		HTLV-2		IND		
	DEV	VAL	DEV	VAL	DEV	VAL	DEV	VAL	
Negative	93	101					**2**	**2**	198
HTLV-1			46	53	**1**				100
HTLV-2					56	43			99
**Total**	93	101	46	53	57	43	2	2	397

Results are expressed by the number of samples, and calculated after applying the algorithm. All samples tested with Multi-HTLV were compared with gold standard results.

DEV, development set; VAL, validation set; IND, Indeterminate.

To further investigate the 4 indeterminate samples obtained, we looked at their serological profiles (Table 4). In both sets, we found negative samples with high reactivity only on the GP21-I/II confirmation antigen. Since this antigen has a very high sensitivity, it was more prudent to consider these samples as indeterminate when only this antigen reacted. The other two indeterminate samples displayed two or three confirmatory antigens with reactivities slightly above the threshold of 10. For this reason, at the first confirmation step, we counted more than one reactive confirmatory antigen, but the logistic regression corrects for this and places them in the indeterminate category (see Fig 3). Despite these four atypical samples, the Multi-HTLV assay provides strong confirmatory performance compared to the currently used WB assays.

**Table 4:** Multi-HTLV intensities for indeterminate samples.

	Gold Standard Results	Multi-HTLV Intensity			Nb antigen with intensity > = 10	LR for confirmation (p)	Final Result
		GP21-I/II CONF	P19-I/II CONF	GP46-I/II CONF			
A) Development	Negative	6,01	**18,77**	**15,49**	2	0,06	IND
	Negative	**36,48**	1,23	1,14	1	0,84	IND
B) Validation	Negative	**42,14**	3,24	1,41	1	0,95	IND
	Negative	**18,92**	**11,81**	**25,84**	2	0,18	IND

(A) Results correspond to indeterminate samples with Multi-HTLV from the development set. The first sample in this group has two positive confirmatory antigens and LR probability (p) less than 0.5, while the second has only one positive GP21-I/II confirmatory antigen but LR probability higher than 0.5 (B) Results correspond to indeterminate samples with Multi-HTLV from the validation set. The first sample in this group has only a positive GP21-I/II confirmatory antigen and LR probability higher than 0.5, while the second has three positive confirmatory antigens but LR probability less than 0.5. CONF, confirmation; Nb, number; LR, Logistic Regression; IND, indeterminate result.

Typing performance showed a strain specificity of 99,03% (94,70–99,83; n = 102/103 correctly typed) for the development set and 100% (96,15–100; n = 96/96) for the validation set. In comparison, the INNO-LIA assay has a strain specificity of 94.17% (87,87–97,30) for the samples in the development set and 95.83% (98,77–98,37) for the samples in the validation set. Among these untyped or falsely typed samples with INNO-LIA, there was a higher proportion of HTLV-2 PCR samples (n = 7/10) (Table 5).

**Table 5:** INNO-LIA scoring results for untyped and falsely typed samples.

	PCR Result	INNO-LIA Scoring results							INNO-LIA Result	Multi-HTLV Result
		GP21-I/II CONF	P19-I/II CONF	P24-I/II CONF	GP46-I/II CONF	P19-I TYPE	GP46-I TYPE	GP46-II TYPE		
A) DEV	HTLV-2	2+	-	2+	-	+/-	-	-	HTLV-1	HTLV-2
	HTLV-2	3+	-	-	2+	-	-	-	HTLV	HTLV-2
	HTLV-2	2+	2+	2+	2+	1+	+/-	+/-	HTLV-1	HTLV-2
	HTLV-2	2+	1+	2+	+/-	-	-	-	HTLV	HTLV-2
	HTLV-1	3+	2+	2+	2+	-	-	-	HTLV	HTLV-2
	HTLV-1	3+	2+	-	1+	-	-	-	HTLV	HTLV-1
B) VAL	HTLV-2	2+	2+	1+	-	-	-	-	HTLV	HTLV-2
	HTLV-2	2+	2+	2+	-	-	-	-	HTLV	HTLV-2
	HTLV-2	2+	-	1+	2+	-	2+	-	HTLV-1	HTLV-2
	HTLV-1	2+	2+	+/-	2+	-	-	-	HTLV	HTLV-1

(A) Results correspond to INNO-LIA untyped and falsely typed samples from the development set. Among the six samples in this group, four have HTLV-2 PCR result and two of them were falsely typed HTLV-1 with INNO-LIA. One sample was non-correctly typed with Multi-HTLV. (B) Results correspond to INNO-LIA untyped and falsely typed samples from the validation set. Among the four samples in this group, three have HTLV-2 PCR result and one of them was falsely typed HTLV-1 with INNO-LIA. All samples were correctly typed with Multi-HTLV. DEV, development set; VAL, validation set; PCR, Polymerase Chain Reaction; CONF, confirmation

In our study, 3 HTLV-1/HCV and 12 HTLV-2/HCV co-infected samples from the HOST cohort were also included. Importantly, all these samples were correctly confirmed and typed with Multi-HTLV (Table 6). We noted that 4 of the samples had a slightly less reactive serological profile. However, the combination of the high sensitivity of the Multi-HTLV and the strength of the algorithm permitted correct confirmation. Thus, co-infection with HTLV/HCV does not seem to have an impact on the test performance.

**Table 6:** Multi-HTLV results for HTLV/HCV co-infected samples.

	PCR Result	Multi-HTLV Intensity						Multi-HTLV Result
		GP21-I/II CONF	P19-I/II CONF	GP46-I/II CONF	P19-I TYPE	GP46-I TYPE	GP46-II TYPE	
A) DEV	HTLV-1	55,3	88,4	98,8	84,6	98,8	5,7	HTLV-1
	HTLV-1	40,6	89,7	101,6	77,8	98,7	1,8	HTLV-1
	HTLV-2	76,0	51,4	88,2	8,2	4,3	81,7	HTLV-2
	HTLV-2	84,9	40,9	89,0	3,7	1,2	90,4	HTLV-2
	HTLV-2	48,3	52,2	96,2	4,2	4,1	90,2	HTLV-2
	HTLV-2	51,2	95,7	108,5	39,3	5,4	100,9	HTLV-2
	HTLV-2	75,6	56,6	42,9	1,6	1,6	40,4	HTLV-2
	HTLV-2	90,0	32,4	14,0	3,1	1,9	20,9	HTLV-2
	HTLV-2	95,4	53,3	16,5	5,7	2,5	11,8	HTLV-2
	HTLV-2	87,6	24,6	9,6	9,2	6,2	9,1	HTLV-2
B) VAL	HTLV-1	60,2	93,4	102,3	80,9	104,4	2,6	HTLV-1
	HTLV-2	80,3	83,1	79,3	5,8	4,1	73,2	HTLV-2
	HTLV-2	90,4	64,9	69,8	6,5	1,9	65,6	HTLV-2
	HTLV-2	84,3	102,9	3,2	15,6	2,8	28,7	HTLV-2
	HTLV-2	77,9	28,6	3,2	3,1	1,9	2,0	HTLV-2

(A) Results correspond to HTLV/HCV co-infected samples from the development set. (B) Results correspond to HTLV/HCV co-infected samples from the validation set.

DEV, development set; VAL, validation set; PCR, Polymerase Chain Reaction; CONF, confirmation

**Fig 5:**
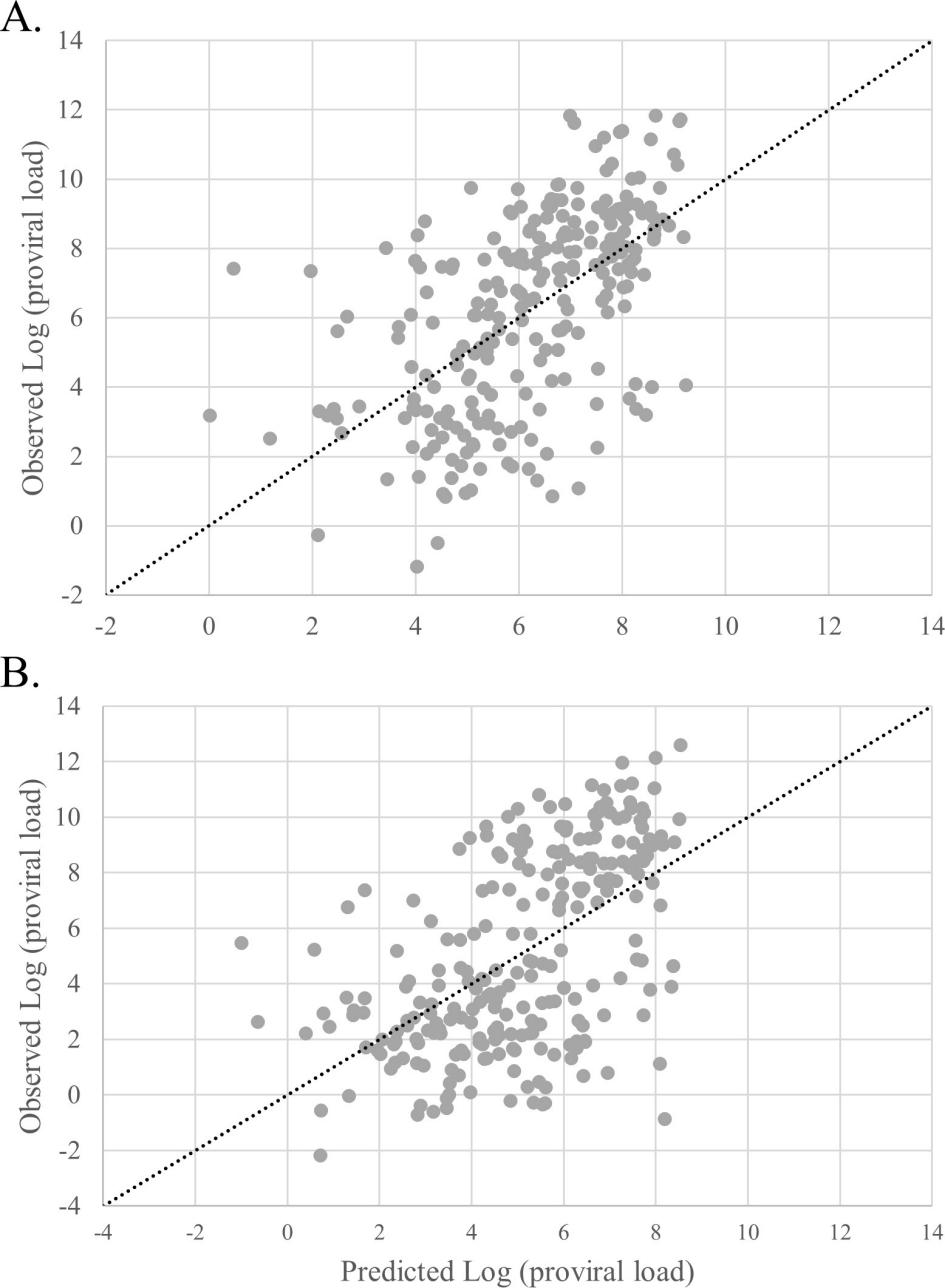
Relationship between observed and predicted logarithm of the proviral load. (A) HTLV-1 samples (N = 246) were plotted by their observed log (PVL) and predicted log (PVL). The linear curve x = y represents the estimated perfect correlation. (B) HTLV-2 samples (N = 245) were plotted by their observed log (PVL) and predicted log (PVL). The linear curve x = y represents the estimated perfect correlation.

Then, we studied the creation of a predictive model for proviral load depending on the serological profile of HTLV positive patients. In 246 HTLV-1 and 245 HTLV-2 samples from the entire HOST cohort, linear and rank correlations between intensities of each antigen of the Multi-HTLV and proviral load were highly significant. Based on these observations, two multivariable models (one for HTLV-1 and one for HTLV-2) were created using the logarithm of the proviral load (log (PVL)) as dependent variable and the antigen intensities as independent variables. Models showed linear Pearson’s correlation coefficients of 0.56 and 0.54 respectively, between observed and predicted proviral load (Fig 5). Although the correlations are strongly significant, the model performance is not sufficient to accurately predict of the proviral load based on antigen reactivities.

Finally, we examined longitudinal patient samples from the HOST cohort using the Multi-HTLV assay. We observed in 35 of 60 patients a certain dynamic in antibody levels between the initial donation and 10–11 years of follow-up. These changes were sometimes visible on the INNO-LIA assay, but measuring the intensities of individual spots with the Multiplex reader provides more accurate values and facilitates visualization of the variations in serological signatures (S4 Appendix; A). However, the variations in antibody levels don’t seem to be associated with variations in proviral load (S4 Appendix; B).

## Discussion

Here we have developed an innovative and high-performance Multi-HTLV assay solving the major limitations associated with the currently used WB confirmatory test. The multiplex assay allows monitoring of different serological profiles for each patient first by visual analysis, and second through a combination of highly specific synthetic antigens as well as a custom-designed high-precision algorithm that ensures the most reliable confirmatory and typing results. The multiplex technology can be performed in any routine laboratory with simple equipment and a small sample volume.

This study targets an important medical need which is to develop a cutting-edge diagnostic tool to improve counselling and reduce viral transmission of HTLV. Over the past 30 years and since the development of the INNO-LIA assay, no significant progress has been made to further improve HTLV diagnostics, thus the current strategy is to simply select the best screening method [29,39]. Our findings on a large number of HTLV samples show that sensitivity correlated 100% with PCR results in the validation sample set, even taking into account HTLV/HCV co-infected samples. Specificity did not reach 100% due to indeterminate results from a low number of PCR-negative samples, but it significantly exceeded the specificity of currently marketed WB assays. [20,40]. The appearance of discordant samples in the development set is related to the training of the logistic regression model. From the antigens reactivities obtained on the samples included in the development set, we have defined logistic regression coefficients in order to obtain the best prediction of PCR gold standard results. However, if several samples display the same antigenic reactivity profile but not the same PCR result, the algorithm will not perfectly match gold standard. Nevertheless, when applied to the validation dataset the developed algorithm, shows high accuracy with no false-positive or false-negative samples.

Despite the imperfect classification of PCR-negative samples with Multi-HTLV, it is preferable to categorize them as indeterminate rather than giving an incorrect result, as they may be subject to further investigation to better understand their status. Furthermore, it would be important to test more co-infected samples and in particular HIV/HTLV samples, which currently cause many difficulties [24].

The Multi-HTLV assay is thus not only a useful tool to accurately diagnose HTLV infection but also a good tool to understand indeterminate samples, which represent a major challenge in HTLV diagnostics, particularly in endemic areas [39]. Indeed, WB indeterminate samples are difficult to resolve in blood bank settings. Several attempts have been made to explain this phenomenon, including samples collected during the seroconversion period, low HTLV-1 proviral load associated with strain gene mutations, other HTLV types (2 to 4), defective HTLV-1 strains, or co-infection with other pathogens [21,24,41]. For instance during seroconversion, antibodies to GP21 appear earlier than those to P24 and P19 [42]. One follow-up study examined indeterminate patient profiles with an isolated GP21-I/II band and found that some patients became HTLV-positive over time suggesting that some patients were in the seroconversion phase at the time of testing [26].

In our study, we also obtained indeterminate results for PCR-negative blood donors, due to an isolated GP21-I/II. Unfortunately, these individuals were not tested for proviral load and follow-up sampling was not performed, making it difficult to understand whether they represent seroconverting cases. For the other indeterminate results, we obtained intensities on antigens other than GP21-I/II that are very close to the minimal intensity value. It is possible that these intensities would vary when the test is repeated and that the final result may change. In a future study, it would be important to track individuals with similar profiles to better understand the role of seroconversion or cross-reactivity. It is also interesting to note, that several studies have identified two serological profiles of WB indeterminate subjects from African and Caribbean populations, called "N" and "HGIP". However, INNO-LIA and PCR tests revealed that these reactivities were probably not caused by HTLV-1 infection but were likely due to cross-reactivity with antigens of other microbial agents, in particular the parasite *Plasmodium falciparum* [22,43]. These studies suggested that a sample is HTLV-positive only if it has reactivity to at least one native P19 and P24 *gag* as well as an *Env* antigen. The multiparametric aspect of the Multi-HTLV assay and its associated confirmation algorithm takes into account the acquisition of at least two reactivities to highly sensitive and specific *Env* and *Gag* antigens and therefore enhances the analysis and provides a more reliable diagnostic of HTLV infection.

Our study also shows that typing of HTLV strains using the Multi-HTLV assay shows promise. Only one sample in the development set was not correctly classified. The proposed method shows higher performance with regard to HTLV type assignment as compared to the INNO-LIA. This would be necessary due to the appearance of new strains in certain endemic areas and in high-risk populations. It is also interesting to note that in our study, INNO-LIA non- or falsely-typed results were related to the HTLV-2 strain. These observations are reminiscent of reported problems with WB, which lacks typing sensitivity particularly for HTLV-2 strains [40]. For INNO-LIA non-typed profiles, PCR is recommended given its higher typing sensitivity [23]. However, it is difficult to completely replace the current confirmatory tests with PCR for the initial testing because HTLV-infected individuals would have to be recalled to obtain fresh sample for additional DNA extraction from peripheral blood cells, and sensitivity to detect low proviral load samples is not sufficient (co-infection or strain-depend) [24,25]. The use of a confirmatory test with high typing sensitivity such as Multi-HTLV would avoid the need for additional confirmatory tests.

Accuracy in distinguishing HTLV strains remains important for prognosis of associated diseases and in cases of some co-infections [44]. For example, HTLV-1 is clearly linked with ATLL but HTLV-2 has not been proven to cause hematologic malignancy. In addition, some studies have shown varying effects on other infectious diseases in the case of co-infection with one of the two HTLV strains. In co-infected individuals, HTLV-1 infection appears to reduce HCV viremia, whereas HTLV-2 increases HCV viral load [44]. In addition, other studies have shown that HIV/HTLV-1 co-infection is associated with rapid progression to the AIDS stage while HTLV-2 plays a protective role [45].

Another medical need related to HTLV infection concerns the understanding of associated diseases because currently no vaccine is available and treatment options for HAM/TSP and ATLL are inadequate [16,46]. The pathogenesis of ATLL is not fully understood and unclear [6]. Interestingly, a correlation between high anti-*env* and proviral DNA levels in HTLV-1 has been observed in patients who develop HAM/TSP compared to asymptomatic patients [46,47]. However, currently there is no serological diagnostic test that differentiates between asymptomatic and symptomatic patients or provides prognostic utility [48]. With the aim of better understanding the type of different serological profiles and the evolution of the infection, we created a model to predict proviral load based on antigens reactivities. The correlation between the observed and predicted proviral load was robust but the performance is not sufficient. More studies are required to investigate whether these models can be used to monitor the proviral load during HTLV infection. However, we observed an interesting dynamic of antibody levels in the majority of the followed-up HTLV individuals. Access to more enriched clinical data from these patients would be very helpful to examine a potential correlation between the evolution of serological signatures the progression to the symptomatic stage.

In summary, our data demonstrate that the Multi-HTLV assay is a novel and reliable assay to detect and type HTLV-infected patients, as well as a research and discovery tool to perform serological profile studies, as recently performed for Chagas disease [49,50]. Since our study includes HTLV-infected individuals, mainly from USA, it is now essential to use larger sample collections that include patients from different regions of the world. It would be of particular interest to test samples from various endemic areas, such as Africa, South America and Australo-Melanesia, that frequently show indeterminate results, and different HTLV-1 genotypes to better assess Multi-HTLV performances. In addition, a comparative analysis of Multi-HTLV and WB’s data would be also important. Finally, it would be useful to extend our study with longitudinal samples over a longer period of time and obtain associated clinical and proviral load data to evaluate prognostic utility. Detailed validation of the Multi-HTLV assay could pave the way to use the test as a monitoring or prediction tool and allow a better counselling and clinical management of the patients.

## Supporting information

S1 Appendix Standards for Reporting Diagnostic accuracy studies (STARD) statement checklist. (DOCX)

S2 Appendix Sample flow chart from the HOST study cohort through Multi-HTLV testing. (DOCX)

S3 Appendix Examples of discordances between visual analysis and algorithm results. In a very few cases, sample can be declared weakly positive by visual analysis, but correctly classified as indetermined with the algorithm. In other cases, samples can be declared both HTLV-1 and HTLV-2 or untyped, but the specific serological profile of the respective type was recognised by the algorithm which assigns the correct result.
(PDF)

S4 Appendix Examples of serological profiles of 3 different patients followed-up overtime. (A) Antigen reactivities obtained with Multi-HTLV and INNO-LIA assays for one HTLV-1 & two HTLV-2 patients. (B) Plots showing comparative evolution of proviral load and the sum of 6 antigens intensities measured by Multi-HTLV for one HTLV-1 and two HTLV-2 patients over 10–11 years.
(PDF)

## References

[1] Gessain A, Cassar O. Epidemiological Aspects and World Distribution of HTLV-1 Infection. Front Microbiol [Internet]. 2012 [cité 16 avr 2020];3. Disponible sur: http://journal.frontiersin.org/article/10.3389/fmicb.2012.00388/abstract 23162541 10.3389/fmicb.2012.00388 PMC3498738

[2] Calattini S, Chevalier SA, Duprez R, Bassot S, Froment A, Mahieux R, Gessain A. Discovery of a new human T-cell lymphotropic virus (HTLV-3) in Central Africa. Retrovirology. 9 mai 2005;2:30. doi: 10.1186/1742-4690-2-30 15882466 PMC1142341

[3] Gallo RC. History of the discoveries of the first human retroviruses: HTLV-1 and HTLV-2. Oncogene. sept 2005;24(39):5926–30. doi: 10.1038/sj.onc.1208980 16155599

[4] Mahieux R, Gessain A. HTLV-3/STLV-3 and HTLV-4 Viruses: Discovery, Epidemiology, Serology and Molecular Aspects. Viruses. 8 juill 2011;3(7):1074–90. doi: 10.3390/v3071074 21994771 PMC3185789

[5] Murphy EL, Cassar O, Gessain A. Estimating the number of HTLV-2 infected persons in the world. Retrovirology. déc 2015;12(S1):O5.

[6] Murphy EL, Lee T, Chafets D, Nass CC, Wang B, Loughlin K, Smith D, HTLV Outcomes Study Investigators. Higher Human T Lymphotropic Virus (HTLV) Provirus Load Is Associated with HTLV-I versus HTLV-II, with HTLV-II Subtype A versus B, and with Male Sex and a History of Blood Transfusion. J Infect Dis. août 2004;190(3):504–10. doi: 10.1086/422398 15243924

[7] Mahieux R, Ibrahim F, Mauclere P, Herve V, Michel P, Tekaia F, Chappey C, Garin B, Van Der Ryst E, Guillemain B, Ledru E, Delaporte E, de The G, Gessain A. Molecular epidemiology of 58 new African human T-cell leukemia virus type 1 (HTLV-1) strains: identification of a new and distinct HTLV-1 molecular subtype in Central Africa and in Pygmies. J Virol. févr 1997;71(2):1317–33. 10.1128/jvi.71.2.1317-1333.1997 PMC191187 8995656

[8] Gessain A. Le rétrovirus humain oncogène HTLV-1: épidémiologie descriptive et moléculaire, origine, évolution et aspects diagnostiques et maladies associées. Bull Société Pathol Exot. août 2011;104(3):167–80. 10.1007/s13149-011-0174-4 21796326

[9] Proietti FA, Carneiro-Proietti ABF, Catalan-Soares BC, Murphy EL. Global epidemiology of HTLV-I infection and associated diseases. Oncogene. sept 2005;24(39):6058–68. doi: 10.1038/sj.onc.1208968 16155612

[10] Schierhout G, McGregor S, Gessain A, Einsiedel L, Martinello M, Kaldor J. Association between HTLV-1 infection and adverse health outcomes: a systematic review and meta-analysis of epidemiological studies. Lancet Infect Dis. janv 2020;20(1):133–43. doi: 10.1016/S1473-3099(19)30402-5 31648940

[11] Paiva A, Casseb J. ORIGIN AND PREVALENCE OF HUMAN T-LYMPHOTROPIC VIRUS TYPE 1 (HTLV-1) AND TYPE 2 (HTLV-2) AMONG INDIGENOUS POPULATIONS IN THE AMERICAS. Rev Inst Med Trop São Paulo. févr 2015;57(1):01–14. doi: 10.1590/S0036-46652015000100001 25651320 PMC4325517

[12] Mauclère P, Meertens L, Afonso P, Plancoulaine S, Filippone C, Betsem E, Calattini S, Froment A, Van Beveren M, de Thé G, Quintana-Murci L, Mahieux R, Gessain A. HTLV-2 in Central Africa: HTLV-2 subtype B strains similar to those found in Amerindian tribes are endemic in Bakola Pygmies from south Cameroon but not in surrounding Bantus and Baka Pygmies. Retrovirology. déc 2011;8(S1):A82, 1742-4690-8-S1-A82.

[13] Araujo A, Hall WW. Human T-lymphotropic virus type II and neurological disease. Ann Neurol. juill 2004;56(1):10–9. doi: 10.1002/ana.20126 15236397

[14] Bartman MT, Kaidarova Z, Hirschkorn D, Sacher RA, Fridey J, Garratty G, Gibble J, Smith JW, Newman B, Yeo AE, Murphy EL, HTLV Outcomes Study (HOST) Investigators. Long-term increases in lymphocytes and platelets in human T-lymphotropic virus type II infection. Blood. 15 nov 2008;112(10):3995–4002. doi: 10.1182/blood-2008-05-155960 18755983 PMC2581993

[15] Biswas HH, Kaidarova Z, Garratty G, Gibble JW, Newman BH, Smith JW, Ziman A, Fridey JL, Sacher RA, Murphy EL. Increased All-Cause and Cancer Mortality in HTLV-II Infection: JAIDS J Acquir Immune Defic Syndr. juill 2010;54(3):290–6. doi: 10.1097/QAI.0b013e3181cc5481 20512047 PMC2891114

[16] Willems L, Hasegawa H, Accolla R, Bangham C, Bazarbachi A, Bertazzoni U, Carneiro-Proietti AB de F, Cheng H, Chieco-Bianchi L, Ciminale V, Coelho-dos-Reis J, Esparza J, Gallo RC, Gessain A, Gotuzzo E, Hall W, Harford J, Hermine O, Jacobson S, Macchi B, Macpherson C, Mahieux R, Matsuoka M, Murphy E, Peloponese J-M, Simon V, Tagaya Y, Taylor GP, Watanabe T, Yamano Y. Reducing the global burden of HTLV-1 infection: An agenda for research and action. Antiviral Res. janv 2017;137:41–8. doi: 10.1016/j.antiviral.2016.10.015 27840202

[17] Ma Y, Zheng S, Wang N, Duan Y, Sun X, Jin J, Zang W, Li M, Wang Y, Zhao G. Epidemiological Analysis of HTLV-1 and HTLV-2 Infection among Different Population in Central China. Vartanian J-P, éditeur. PLoS ONE. 24 juin 2013;8(6):e66795. doi: 10.1371/journal.pone.0066795 23826136 PMC3691312

[18] Zihlmann KF, de Alvarenga AT, Casseb J. Living Invisible: HTLV-1-Infected Persons and the Lack of Care in Public Health. Kashanchi F, éditeur. PLoS Negl Trop Dis. 12 juin 2012;6(6):e1705. doi: 10.1371/journal.pntd.0001705 22720112 PMC3373594

[19] da Silva Brito V, Santos FLN, Gonçalves NLS, Araujo THA, Nascimento DSV, Pereira FM, Boa-Sorte NCA, Grassi MFR, Caterino-de-Araujo A, Galvão-Castro B. Performance of Commercially Available Serological Screening Tests for Human T-Cell Lymphotropic Virus Infection in Brazil. Tang Y-W, éditeur. J Clin Microbiol. 19 sept 2018;56(12):e00961–18, /jcm/56/12/e00961-18.atom. doi: 10.1128/JCM.00961-18 30232131 PMC6258847

[20] Campos KR, Santos FLN, da Silva Brito V, Gonçalves NLS, Araujo THA, Galvão-Castro B, Caterino-de-Araujo A. Line Immunoassay for Confirmation and Discrimination of Human T-Cell Lymphotropic Virus Infections in Inconclusive Western Blot Serum Samples from Brazil. Tang Y-W, éditeur. J Clin Microbiol. 9 oct 2019;58(1):e01384–19, /jcm/58/1/JCM.01384-19.atom. doi: 10.1128/JCM.01384-19 31597749 PMC6935901

[21] Kuramitsu M, Sekizuka T, Yamochi T, Firouzi S, Sato T, Umeki K, Sasaki D, Hasegawa H, Kubota R, Sobata R, Matsumoto C, Kaneko N, Momose H, Araki K, Saito M, Nosaka K, Utsunomiya A, Koh K-R, Ogata M, Uchimaru K, Iwanaga M, Sagara Y, Yamano Y, Okayama A, Miura K, Satake M, Saito S, Itabashi K, Yamaguchi K, Kuroda M, Watanabe T, Okuma K, Hamaguchi I. Proviral Features of Human T Cell Leukemia Virus Type 1 in Carriers with Indeterminate Western Blot Analysis Results. Caliendo AM, éditeur. J Clin Microbiol. sept 2017;55(9):2838–49. doi: 10.1128/JCM.00659-17 28701419 PMC5648719

[22] Filippone C, Bassot S, Betsem E, Tortevoye P, Guillotte M, Mercereau-Puijalon O, Plancoulaine S, Calattini S, Gessain A. A New and Frequent Human T-Cell Leukemia Virus Indeterminate Western Blot Pattern: Epidemiological Determinants and PCR Results in Central African Inhabitants. J Clin Microbiol. 1 mai 2012;50(5):1663–72. doi: 10.1128/JCM.06540-11 22403426 PMC3347141

[23] Andrade RG, Ribeiro MA, Namen-Lopes MSS, Silva SMN, Basques FV, Ribas JG, Carneiro-Proietti AB de F, Martins ML. Evaluation of the use of real-time PCR for human T cell lymphotropic virus 1 and 2 as a confirmatory test in screening for blood donors. Rev Soc Bras Med Trop. avr 2010;43(2):111–5. doi: 10.1590/s0037-86822010000200001 20464136

[24] Campos KR, Gonçalves MG, Costa NA, Caterino-de-Araujo A. Comparative performances of serologic and molecular assays for detecting human T lymphotropic virus type 1 and type 2 (HTLV-1 and HTLV-2) in patients infected with human immunodeficiency virus type 1 (HIV-1). Braz J Infect Dis. mai 2017;21(3):297–305. doi: 10.1016/j.bjid.2017.02.005 28343818 PMC9428028

[25] Arruda BC, Lira RA, Loureiro P, Brandão L, Souza P, Souza WV, Gomes YM. Evaluation of real time PCR technique to diagnosis of human T-lymphotropic virus type I (HTLV-I) in patients in the Hematologia da Fundação Hemope Hospital, in Northeastern Brazil. Rev Bras Hematol E Hemoter [Internet]. oct 2008 [cité 15 avr 2020];30(5). Disponible sur: http://www.scielo.br/scielo.php?script=sci_arttext&pid=S1516-84842008000500011&lng=en&nrm=iso&tlng=en

[26] Ji H, Chang L, Yan Y, Jiang X, Sun H, Guo F, Wang L. A Strategy for Screening and Confirmation of HTLV-1/2 Infections in Low-Endemic Areas. Front Microbiol. 3 juin 2020;11:1151. doi: 10.3389/fmicb.2020.01151 32582093 PMC7283491

[27] Sabino EC, Zrein M, Taborda CP, Otani MM, Ribeiro-Dos-Santos G, Sáez-Alquézar A. Evaluation of the INNO-LIA HTLV I/II Assay for Confirmation of Human T-Cell Leukemia Virus-Reactive Sera in Blood Bank Donations. J Clin Microbiol. 1999;37(5):1324–8. doi: 10.1128/JCM.37.5.1324-1328.1999 10203479 PMC84764

[28] Zrein M, Louwagie J, Boeykens H, Govers L, Hendrickx G, Bosman F, Sablon E, Demarquilly C, Boniface M, Saman E. Assessment of a new immunoassay for serological confirmation and discrimination of human T-cell lymphotropic virus infections. Clin Diagn Lab Immunol. janv 1998;5(1):45–9. doi: 10.1128/CDLI.5.1.45-49.1998 9455879 PMC121390

[29] Okuma K, Kuramitsu M, Niwa T, Taniguchi T, Masaki Y, Ueda G, Matsumoto C, Sobata R, Sagara Y, Nakamura H, Satake M, Miura K, Fuchi N, Masuzaki H, Okayama A, Umeki K, Yamano Y, Sato T, Iwanaga M, Uchimaru K, Nakashima M, Utsunomiya A, Kubota R, Ishitsuka K, Hasegawa H, Sasaki D, Koh K-R, Taki M, Nosaka K, Ogata M, Naruse I, Kaneko N, Okajima S, Tezuka K, Ikebe E, Matsuoka S, Itabashi K, Saito S, Watanabe T, Hamaguchi I. Establishment of a novel diagnostic test algorithm for human T-cell leukemia virus type 1 infection with line immunoassay replacement of western blotting: a collaborative study for performance evaluation of diagnostic assays in Japan. Retrovirology. déc 2020;17(1):26. doi: 10.1186/s12977-020-00534-0 32831150 PMC7444053

[30] Campos KR, Caterino-de-Araujo A. Provirus Mutations of Human T-Lymphotropic Virus 1 and 2 (HTLV-1 and HTLV-2) in HIV-1-Coinfected Individuals. Ono A, éditeur. mSphere [Internet]. 28 oct 2020 [cité 11 juin 2021];5(5). Disponible sur: https://journals.asm.org/doi/10.1128/mSphere.00923-20 10.1128/mSphere.00923-20 PMC7529439 32999083

[31] Busch MP, Laycock M, Kleinman SH, Wages JW, Calabro M, Kaplan JE, Khabbaz RF, Hollingsworth CG. Accuracy of supplementary serologic testing for human T-lymphotropic virus types I and II in US blood donors. Retrovirus Epidemiology Donor Study. Blood. 15 févr 1994;83(4):1143–8. 8111054

[32] Murphy EL. Increased Incidence of Infectious Diseases During Prospective Follow-up of Human T-Lymphotropic Virus Type II–and I–Infected Blood Donors. Arch Intern Med. 12 juill 1999;159(13):1485. doi: 10.1001/archinte.159.13.1485 10399901

[33] Murphy EL, Glynn SA, Fridey J, Sacher RA, Smith JW, Wright DJ, Newman B, Gibble JW, Ameti DI, Nass CC, Schreiber GB, Nemo GJ, for the Retrovirus Epidemiology Donor Study (REDS) Study Group. Increased Prevalence of Infectious Diseases and Other Adverse Outcomes in Human T Lymphotropic Virus Types I- and II-Infected Blood Donors. J Infect Dis. déc 1997;176(6):1468–75. doi: 10.1086/514143 9395356

[34] Lee T-H, Chafets DM, Busch MP, Murphy EL. Quantitation of HTLV-I and II proviral load using real-time quantitative PCR with SYBR Green chemistry. J Clin Virol. déc 2004;31(4):275–82. doi: 10.1016/j.jcv.2004.05.016 15494269

[35] Granjon E, Dichtel-Danjoy M-L, Saba E, Sabino E, Campos de Oliveira L, Zrein M. Development of a Novel Multiplex Immunoassay Multi-cruzi for the Serological Confirmation of Chagas Disease. PLoS Negl Trop Dis. 1 avr 2016;10(4):e0004596. doi: 10.1371/journal.pntd.0004596 27035146 PMC4818036

[36] Martins ML, Santos AC da S, Namen-Lopes MS, Barbosa-Stancioli EF, Utsch DG, Carneiro-Proietti AB de F. Long-term serological follow-up of blood donors with an HTLV-Indeterminate Western Blot: Antibody Profile of Seroconverters and Individuals With False Reactions. J Med Virol. 1 sept 2010;82(10):1746–53. doi: 10.1002/jmv.21881 20827773

[37] Gelman A, Jakulin A, Pittau MG, Su Y-S. A weakly informative default prior distribution for logistic and other regression models. Ann Appl Stat. déc 2008;2(4):1360–83.

[38] Akaike H. A new look at the statistical model identification. IEEE Trans Autom Control. déc 1974;19(6):716–23.

[39] World Health Organization. Human T-Lymphotropic Virus Type 1: Technical Report [Internet]. 2021 févr [cité 10 juin 2021]. Report No.: 978-92-4-002022-1. Disponible sur: https://www.who.int/publications/i/item/9789240020221

[40] Olah I, Fukumori LMI, Smid J, de Oliveira ACP, Duarte AJS, Casseb J. Neither molecular diversity of the envelope, immunosuppression status, nor proviral load causes indeterminate HTLV western blot profiles in samples from human T-cell lymphotropic virus type 2 (HTLV-2)-infected individuals. J Med Virol. mai 2010;82(5):837–42. doi: 10.1002/jmv.21718 20336719

[41] Cánepa C, Salido J, Ruggieri M, Fraile S, Pataccini G, Berini C, Biglione M. Low Proviral Load is Associated with Indeterminate Western Blot Patterns in Human T-Cell Lymphotropic Virus Type 1 Infected Individuals: Could Punctual Mutations be Related? Viruses. 28 oct 2015;7(11):5643–58. doi: 10.3390/v7112897 26516904 PMC4664970

[42] Manns A, Murphy EL, Wilks R, Haynes G, Figueroa JP, Hanchard B, Barnett M, Drummond J, Waters D, Cerney M. Detection of early human T-cell lymphotropic virus type I antibody patterns during seroconversion among transfusion recipients. Blood. 15 févr 1991;77(4):896–905. 1993227

[43] Rouet F, Meertens L, Courouble G, Herrmann-Storck C, Pabingui R, Chancerel B, Abid A, Strobel M, Mauclere P, Gessain A. Serological, Epidemiological, and Molecular Differences between Human T-Cell Lymphotropic Virus Type 1 (HTLV-1)-Seropositive Healthy Carriers and Persons with HTLV-I Gag Indeterminate Western Blot Patterns from the Caribbean. J Clin Microbiol. 1 avr 2001;39(4):1247–53. doi: 10.1128/JCM.39.4.1247-1253.2001 11283036 PMC87919

[44] Caterino-de-Araujo A, Alves FA, Campos KR, Lemos MF, Moreira RC. Making the invisible visible: searching for human T-cell lymphotropic virus types 1 and 2 (HTLV-1 and HTLV-2) in Brazilian patients with viral hepatitis B and C. Mem Inst Oswaldo Cruz. févr 2018;113(2):130–4. doi: 10.1590/0074-02760170307 29236927 PMC5722269

[45] Pilotti E, Bianchi MV, De Maria A, Bozzano F, Romanelli MG, Bertazzoni U, Casoli C. HTLV-1/-2 and HIV-1 co-infections: retroviral interference on host immune status. Front Microbiol [Internet]. 2013 [cité 24 nov 2020];4. Disponible sur: http://journal.frontiersin.org/article/10.3389/fmicb.2013.00372/abstract 24391628 10.3389/fmicb.2013.00372 PMC3870298

[46] Nozuma S, Jacobson S. Neuroimmunology of Human T-Lymphotropic Virus Type 1-Associated Myelopathy/Tropical Spastic Paraparesis. Front Microbiol. 24 avr 2019;10:885. doi: 10.3389/fmicb.2019.00885 31105674 PMC6492533

[47] Kubota R, Kawanishi T, Matsubara H, Manns A, Jacobson S. HTLV-I specific IFN-γ+ CD8+ lymphocytes correlate with the proviral load in peripheral blood of infected individuals. J Neuroimmunol. janv 2000;102(2):208–15. doi: 10.1016/s0165-5728(99)00175-7 10636490

[48] Burbelo PD, Meoli E, Leahy HP, Graham J, Yao K, Oh U, Janik JE, Mahieux R, Kashanchi F, Iadarola MJ, Jacobson S. Anti-HTLV antibody profiling reveals an antibody signature for HTLV-I-Associated Myelopathy/Tropical Spastic Paraparesis (HAM/TSP). Retrovirology. 2008;5(1):96. 18937847 10.1186/1742-4690-5-96 PMC2580768

[49] Zrein M, Granjon E, Gueyffier L, Caillaudeau J, Liehl P, Pottel H, Cardoso CS, Oliveira CDL, de Oliveira LC, Lee T-H, Ferreira AM, Ribeiro ALP, Busch MP, Sabino EC. A novel antibody surrogate biomarker to monitor parasite persistence in Trypanosoma cruzi-infected patients. PLoS Negl Trop Dis. 9 févr 2018;12(2):e0006226. doi: 10.1371/journal.pntd.0006226 29425201 PMC5823467

[50] Jurado Medina L, Chassaing E, Ballering G, Gonzalez N, Marqué L, Liehl P, Pottel H, de Boer J, Chatelain E, Zrein M, Altcheh J. Prediction of Parasitological Cure in Trypanosoma cruzi-infected Children using a Novel Multiplex Serological Approach: an observational retrospective cohort study. The Lancet Infectious Diseases. Accepted;</References> 10.1016/S1473-3099(20)30729-5 33836157

