# Supplementary material for: A novel high performing multiplex immunoassay Multi-HTLV for serological confirmation and typing of HTLV infections
Source: PLoS Negl Trop Dis. 2021 Nov 1;15(11):e0009925. doi: 10.1371/journal.pntd.0009925 (PMC8584783; doi:10.1371/journal.pntd.0009925)
Supplement: S1 Appendix — (DOCX) [file pntd.0009925.s001.docx]

|  | **Section & Topic** | **No** | **Item** | **Reported on page #** |
| --- | --- | --- | --- | --- |
|  |  |  |  |  |
|  | **ABSTRACT** | **1** | Identification as a study of diagnostic accuracy using at least one measure of accuracy  (such as sensitivity, specificity, predictive values, or AUC) | 2 (Abstract) |
|  |  | **2** | Contains summary of study aim, methods for assay development and validation, the results obtained and conclusions. | 2, 3 |
|  | **INTRODUCTION** | **3** | Scientific and clinical background, including the intended use and clinical role of the index test.  Introduction of the infectious disease, explanation of the problems related with poor diagnostic performance and link with current methods | 3, 4, 5 |
|  |  | **4** | Study objectives and hypotheses: the objective of the project is to improve the performance of the diagnosis and to adress the hypotheses stated in the introduction | 5, 6 |
|  | **METHODS**  *Study design* | **5** | Whether data collection was planned before the index test and reference standard  were performed (prospective study) or after (retrospective study) | 6, 7, 8 |
|  | *Participants* | **6** | Eligibility criteria | 7, 8, Fig 1 |
|  |  | **7** | Identification of eligible participants from screening test result, symptoms and physical examinations. | 7, 8, Table 1 |
|  |  | **8** | The location and dates for identification of eligible participants are available | 7, 8 |
|  |  | **9** | Participants of HOST cohort formed a convenient series  Participants of EFS and Trans-Hit Bio formed random series | 7, 8 |
|  | *Test methods* | **10a** | Index test, in sufficient detail to allow replication | 9, 10, 11, Fig 2, Table 2 |
|  |  | **10b** | Reference standard, in sufficient detail to allow replication | Not applicable |
|  |  | **11** | Rationale for choosing the reference standard (if alternatives exist) | 12 |
|  |  | **12a** | Definition of and rationale for test positivity cut-offs or result categories  of the index test, distinguishing pre-specified from exploratory | 13, 14, Fig 3 |
|  |  | **12b** | Definition of and rationale for test positivity cut-offs or result categories  of the reference standard, distinguishing pre-specified from exploratory | Not applicable |
|  |  | **13a** | Whether clinical information and reference standard results were available  to the performers/readers of the index test | Not applicable |
|  |  | **13b** | Whether clinical information and index test results were available  to the assessors of the reference standard | Not applicable |
|  | *Analysis* | **14** | Comparison of diagnostic accuracy based on calculation of sensitivity and specificity | 14 |
|  |  | **15** | How indeterminate index test or reference standard results were handled | 14 |
|  |  | **16** | How missing data on the index test and reference standard were handled | Not applicable |
|  |  | **17** | Any analyses of variability in diagnostic accuracy, distinguishing pre-specified from exploratory | Not applicable |
|  |  | **18** | Sample size was determined randomly. | 12 |
|  | **RESULTS** *Participants* | **19** | Flow of participants, using a diagram | Fig 1, S2 appendix |
|  |  | **20** | Baseline demographic and clinical characteristics of participants | Not applicable |
|  |  | **21a** | Distribution of severity of disease in those with the target condition | Not applicable |
|  |  | **21b** | Distribution of alternative diagnoses in those without the target condition | Not applicable |
|  |  | **22** | Time interval and any clinical interventions between index test and reference standard | Not applicable |
|  | *Test results* | **23** | Cross tabulation of the index test results (or their distribution)  by the results of the reference standard | Table 3 |
|  |  | **24** | Estimates of diagnostic accuracy and their precision (such as 95% confidence intervals) | 15, 18 |
|  |  | **25** | Any adverse events from performing the index test or the reference standard | Not applicable |
|  | **DISCUSSION** | **26** | Study limitations, including sources of potential bias, statistical uncertainty, and generalisability | 22, 23, 24, 25, 26 |
|  |  | **27** | Implications for practice, including the intended use and clinical role of the index test | 22, 23, 24, 25, 26 |
|  | **OTHER INFORMATION** | **28** | Registration number and name of registry | Not applicable |
|  |  | **29** | Where the full study protocol can be accessed | Not applicable |
|  |  | **30** | Sources of funding and other support; role of funders | Not applicable |
|  |  |  |  |  |
