# Supplementary figures and images for: A novel high performing multiplex immunoassay Multi-HTLV for serological confirmation and typing of HTLV infections
Source: PLoS Negl Trop Dis. 2021 Nov 1;15(11):e0009925. doi: 10.1371/journal.pntd.0009925 (PMC8584783; doi:10.1371/journal.pntd.0009925)

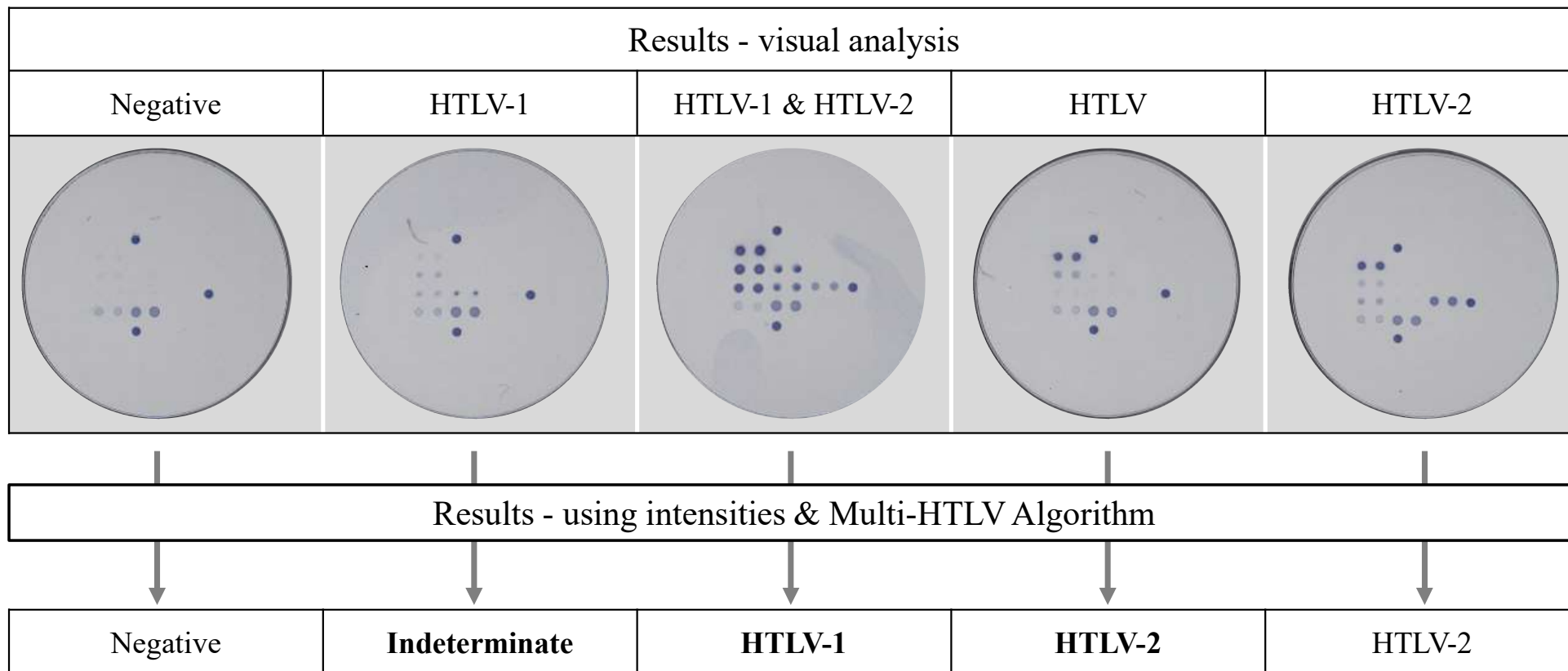

Supplement: S3 Appendix — In a very few cases, sample can be declared weakly positive by visual analysis, but correctly classified as indetermined with the algorithm. In other cases, samples can be declared both HTLV-1 and HTLV-2 or untyped, but the specific serological profile of the respective type was recognised by the algorithm which assigns the correct result. (PDF) [file pntd.0009925.s003.pdf]
