# Supplementary material for: A novel high performing multiplex immunoassay Multi-HTLV for serological confirmation and typing of HTLV infections
Source: PLoS Negl Trop Dis. 2021 Nov 1;15(11):e0009925. doi: 10.1371/journal.pntd.0009925 (PMC8584783; doi:10.1371/journal.pntd.0009925)

A.

Patient ID	Sampling Date	PCR Result	Proviral Load	INNO-LIA Scoring							Sum INNO-LIA scoring	INNO-LIA Result	Multi-HTLV intensity						Sum Multi-HTLV intensities	Multi-HTLV Result
				GP21-I/II CONF	P19-I/II CONF	P24-I/II CONF	GP46-I/II CONF	P19-I TYPE	GP46-I TYPE	GP46-II TYPE			GP21-I/II CONF	P19-I/II CONF	GP46-I/II CONF	P19-I TYPE	GP46-I TYPE	GP46-II TYPE		
108	19/06/91	HTLV-1	2172	3	2	2	2	0	3	0	12	HTLV-1	47,9	83,6	95,4	48,1	99,0	1,0	375,0	HTLV-1
	16/04/93	HTLV-1	6589	3	3	2	2	0	3	0,5	13,5	HTLV-1	75,3	101,5	87,7	60,8	91,1	0,4	416,8	HTLV-1
	10/04/95	HTLV-1	9962	3	3	3	2	0	3	0,5	14,5	HTLV-1	56,5	95,3	93,5	87,1	92,2	3,0	427,6	HTLV-1
	04/02/98	HTLV-1	17110	3	3	3	2	0	3	0	14	HTLV-1	80,4	90,2	83,1	64,4	87,3	1,5	406,9	HTLV-1
	07/06/00	HTLV-1	8709	4	3	3	3	0	3	0	16	HTLV-1	80,3	85,5	83,2	39,1	83,7	3,4	375,1	HTLV-1
	18/09/02	HTLV-1	8200	4	3	3	3	0	3	0	16	HTLV-1	83,9	86,6	81,2	24,5	82,0	3,7	361,9	HTLV-1
572	01/07/92	HTLV-2	1	3	0	2	2	0	0	2	9	HTLV-2	51,4	29,8	53,3	4,9	3,8	47,6	190,9	HTLV-2
	21/10/93	HTLV-2	24	3	0	2	2	0	0	2	9	HTLV-2	85,0	12,7	39,3	0,7	2,9	38,9	179,5	HTLV-2
	04/04/96	HTLV-2	2042	3	0,5	2	2	0	0	2	9,5	HTLV-2	82,0	22,4	98,3	2,4	3,1	93,8	302,1	HTLV-2
	04/08/98	HTLV-2	7	3	0,5	2	2	0	0	3	10,5	HTLV-2	85,2	28,0	96,1	1,9	3,2	89,9	304,4	HTLV-2
	10/02/00	HTLV-2	35576	3	0	2	2	0	0	2	9	HTLV-2	94,8	26,8	96,3	2,1	2,6	91,4	314,0	HTLV-2
	22/07/02	HTLV-2	184	3	0	2	2	0	0	2	9	HTLV-2	91,1	33,3	99,3	5,6	4,2	95,3	328,8	HTLV-2
1204	23/01/91	HTLV-2	32	3	2	2	2	0	0	2	11	HTLV-2	75,6	56,6	42,9	1,6	1,6	40,4	218,7	HTLV-2
	02/10/93	HTLV-2	29	3	2	2	2	0	0	2	11	HTLV-2	81,9	35,2	40,8	1,4	1,8	41,6	202,6	HTLV-2
	07/10/95	HTLV-2	2	3	2	2	2	0	0	2	11	HTLV-2	88,3	52,7	56,8	7,0	2,6	63,9	271,2	HTLV-2
	03/05/98	HTLV-2	333	3	2	2	2	0	0	2	11	HTLV-2	83,0	44,8	39,7	7,0	4,5	41,0	219,9	HTLV-2
	02/12/02	HTLV-2	18	3	2	2	2	0	0	2	11	HTLV-2	93,7	31,1	16,1	4,6	3,7	40,0	189,3	HTLV-2

B.

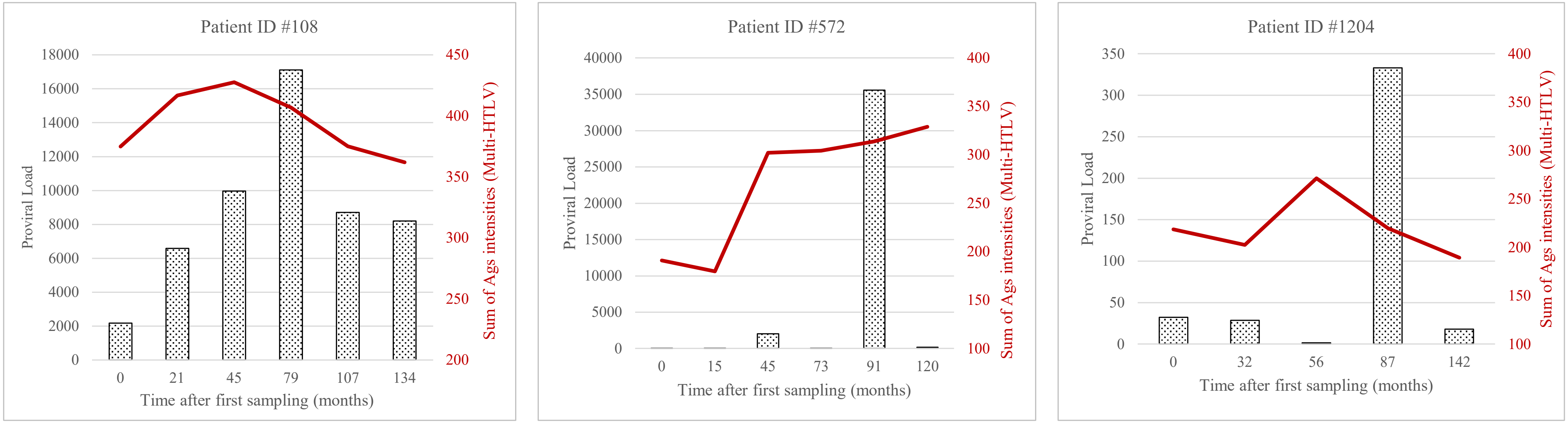

Supplement: S4 Appendix — (A) Antigen reactivities obtained with Multi-HTLV and INNO-LIA assays for one HTLV-1 & two HTLV-2 patients. (B) Plots showing comparative evolution of proviral load and the sum of 6 antigens intensities measured by Multi-HTLV for one HTLV-1 and two HTLV-2 patients over 10–11 years. (PDF) [file pntd.0009925.s004.pdf]
