# Supplementary material for: A novel high performing multiplex immunoassay Multi-HTLV for serological confirmation and typing of HTLV infections
Source: PLoS Negl Trop Dis. 2021 Nov 1;15(11):e0009925. doi: 10.1371/journal.pntd.0009925 (PMC8584783; doi:10.1371/journal.pntd.0009925)
Supplement: Supplementary file 4 [file pntd.0009925.s002.docx]

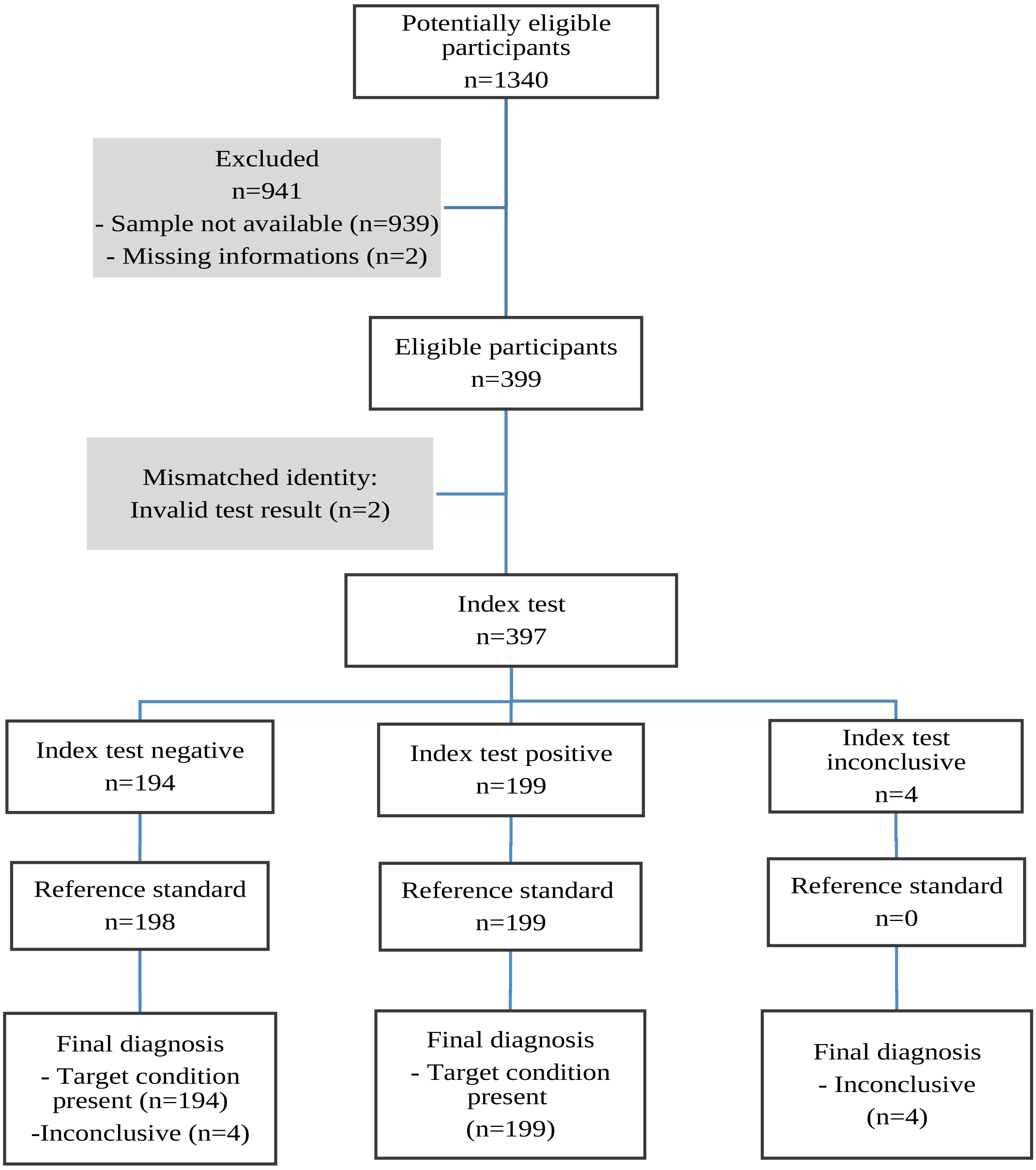
Potentially eligible participants
n=1340
Excluded
n=941
- Sample not available (n=939)
- Missing informations (n=2)
Eligible participants
n=399
Mismatched identity:
Invalid test result (n=2)
Index test
n=397
Index test negative
n=194
Index test positive
n=199
Index test inconclusive
n=4
Reference standard
n=198
Reference standard
n=199
Reference standard
n=0
Final diagnosis
- Target condition present (n=194)
-Inconclusive (n=4)
Final diagnosis
- Target condition present
(n=199)
Final diagnosis
- Inconclusive
(n=4)
